# C19, a C-terminal peptide of CKLF1, decreases inflammation and proliferation of dermal capillaries in psoriasis

**DOI:** 10.1038/s41598-017-13799-x

**Published:** 2017-10-24

**Authors:** Yi Zheng, Yixuan Wang, Xuan Zhang, Yaqi Tan, Shiguang Peng, Le Chen, Yanling He

**Affiliations:** Department of Dermatology, Beijing Chaoyang Hospital Affiliated to Capital Medical University, Beijing, China

## Abstract

Psoriasis is a chronic inflammatory autoimmune disease with undefined etiology. Chemokine-like factor 1 (CKLF1), a human cytokine that is a functional ligand for CCR4, displays chemotactic activities in a wide spectrum of leukocytes and plays an important role in psoriasis development. In previous study, our laboratory found that the expression of CKLF1 increased in psoriatic lesions. C19 as a CKLF1’s C-terminal peptide has been reported to exert inhibitory effects on a variety of diseases. However, the protective roles of C19 in endothelial cells proliferation and inflammatory cells chemotaxis remain elusive in psoriasis. In this study we examined the protective effect of C19 on both the cellular model and the animal model. The effects of C19 on endothelial cells proliferation and inflammatory cells chemotaxis were investigated in cultured human umbilical vein endothelial cells (HUVECs) and imiquimod-induced psoriasiform inflammation of BALB/c mice based on techniques including immunohistochemical analysis, quantitative real-time PCR (qRT-PCR), western blot, transwell, and EdU assay. This study shows that CKLF1-C19 significantly protects against psoriasis by inhibiting the infiltration of inflammatory cells and proliferation of microvascular cells, possibly via inhibiting MAPK pathways.

## Introduction

Psoriasis is an inflammatory skin disease mediated by the cells and molecules of both the innate and adaptive immune system. It is characterized by epidermal hyper proliferation, increase in keratin expression, recruitment of T cells and changes in the endothelial vascular system^1^. The dysfunction of immune system has been known to be an important factor in the pathogenesis of psoriasis, meanwhile, strong evidences also indicate that microvascular changes, including dilatation, tortuosity, increased permeability, and endothelial cell proliferation within the venous limb of capillaries in the dermal papillae, contribute to the disease progress^2,3^. CD4 lymphocytes of Th1, Th17 migrate to the skin, evoke the inflammation through their activation and cytokine-and chemokine-mediated interaction with other cells. According to current knowledge, TNF-α and IFN-γ are the primary inflammation factors as they induce the synthesis of secondary inflammatory cytokines and chemokines^4^.

Chemokines, which were originally identified as chemotactic factors for leukocytes, constituted a large family of structurally related cytokines^5,6^, a family of approximately 50 chemoattractant cytokines, have been divided into four subfamilies: CXC, CC, C and CX3C. The expression and function of chemokines have been intensively investigated in inflammatory and allergic responses in peripheral organs such as the lung and kidney^7,8^. It has been confirmed that in psoriasis the recruitment of leukocytes to the skin is mediated by the chemokine and chemotactic cytokine network^9^. Therefore, chemokines are promising targets for development of novel and effective therapeutics for psoriasis.

Chemokine-like factor 1 (CKLF1) is a novel human cytokine of the CC chemokine gene family, firstly discovered through isolation from phytohemagglutinin-stimulated U937 cells, and a novel functional ligand for the CCR4^10^, which prevalently expressed on T cells^11^. CKLF1 displays chemotactic activities in a wide spectrum of leukocytes and neutrophils. The expression of CKLF1 is up-regulated in various inflammatory and autoimmune diseases^12^. It also enhances proliferation of bone marrow cells *in vitro* and stimulates the effect of immature dendritic cells on T cell proliferation and IFN-γ production^13,14^. CKLF1 contains at least two secreted subforms located at its C-terminal part, termed as C19, which is obtained from secreted CKLF1 stably expressed in Drosophila S2 cells, and C27. C19 has weaker chemotactic activity than those induced by CKLF1 or C27, which is abolished by pertussis toxin, and inhibited by an antagonist of CCR4. C19 inhibits chemotaxis induced by CKLF1 or CCL17 (TARC) or CCL22 (MDC). These results confirm that the secreted peptides C19 has functional activation via CCR4^15^. In addition, C19 protects the brain against ischemia by decreasing production of mediators as TNF-α, IL-1β, and IL-8 to reduce neutrophil infiltration to ischemic areas, possibly via inhibiting the MAPK pathways in rats^16^. Furthermore, C19 inhibits neointima formation *in vivo* and *in vitro*, indicating its protective role in restenosis^17^. Our previous research demonstrated that the expression of CKLF1, induced by TNF-α or psoriasis serum in human umbilical vein endothelial cells (HUVECs), increased in psoriatic lesions^18^.

In present study, we first found that C19 protected against psoriasis by inhibiting the proliferation of microvascular endothelial and inflammatory cells infiltration via MAPK pathways.

## Results

### Distribution of CCL17 in lesional psoriatic skin and normal human skin

**Figure 1:**
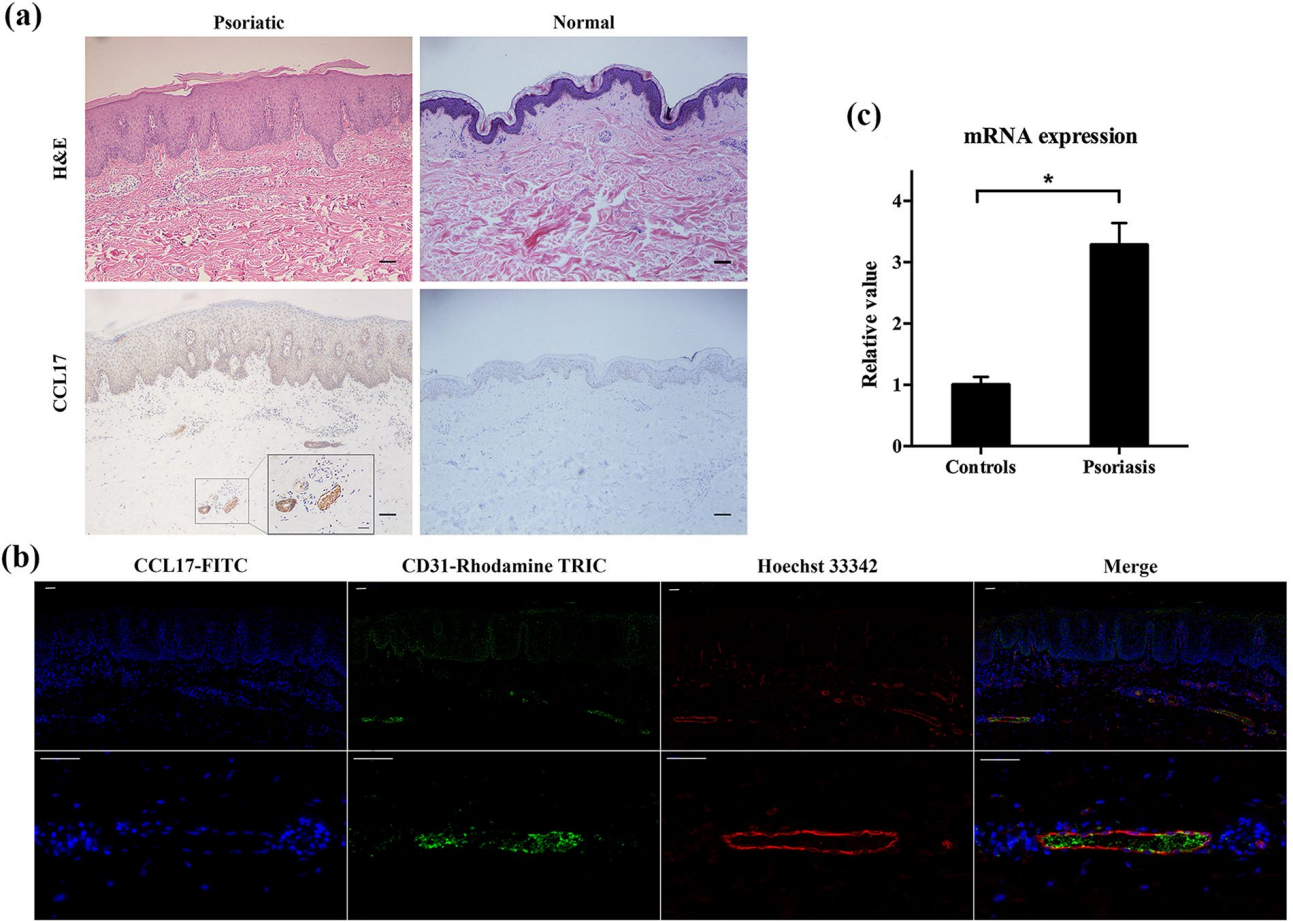
CCL17 proteins was strongly expressed in psoriatic lesions. Paraffin sections of skin tissues were stained by H&E or immunohistochemistry for CCL17. (**a**) Compared with normal controls, psoriatic skins exhibited stronger staining of CCL17 in both epidermis and dermal microvessels. Scale bar = 100 μm. (**b**) Frozen sections were also analyzed for CCL17 and CD31 expression. By immunofluorescence, top panel showed the expression of CCL17 (green), CD31 (red), and nuclei (blue). Bottom panel represented the magnified dermal microvessels that expressed intense CCL17 and CD31. Scale bar = 50 μm. (**c**) qRT-PCR showed high expression of CCR4 mRNA in PBMCs of psoriasis patients compared with that of healthy individuals. Number of tissue samples were 15 (psoriatic) and 5 (normal), respectively. *p < 0.05.

By using hematoxylin and eosin (H&E) stain, we observed the epidermis hyperproliferation, inflammatory cell accumulation, and dilation of dermal papillary blood vessels. We confirmed that there were more CCL17 expressing cells in plaque psoriasis than in normal skin using immunohistochemistry (Fig. 1a, Supplementary Fig. S1). In the former, diffuse expression of CCL17 was observed throughout the epidermis and in the dermal microvessels that were composed of endothelial cells. However, little staining of CCL17 was seen in normal tissues. The strong expression of CCL17 in psoriasis skins were also reflected by immunofluorescence that showed intense CCL17 staining in dermal microvessels (Fig. 1b).

The gene expression of CCR4 in the peripheral blood mononuclear cells (PBMCs) of psoriasis patients was detected by quantitative real-time PCR (qRT-PCR). We found a significant up-regulation of CCR4 mRNA in the PBMCs of psoriasis patients compared with healthy individuals (Fig. 1c).

### TNF-α induced expression of chemokines in HUVECs

**Figure 2:**
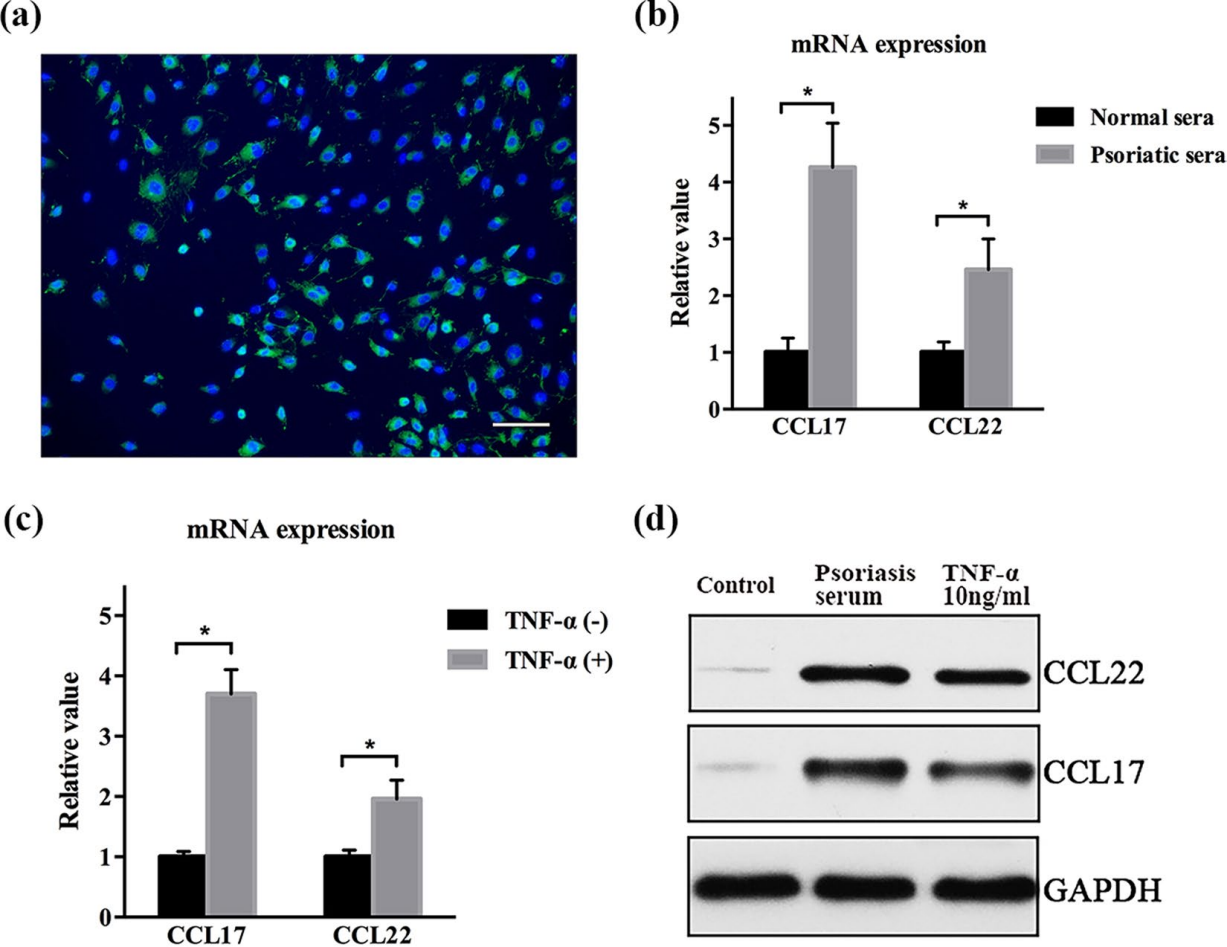
Psoriatic inflammation enhanced CCL17 and CCL22 expression by HUVECs. (**a**) Primary HUVECs exhibited strong factor VIII staining (green) by immunofluorescence. Nuclei were stained by 4′,6-diamidino-2-phenylindole (blue). Scale bar = 100 μm. (**b**) Serum of psoriasis patients and (**c**) TNF-α displayed enhancement effect on mRNA expression of CCL17 and CCL22 by HUVECs, which was consistent with the protein expression of CCL17 and CCL22 (**d**). Data were obtained from three independent experiments. *p < 0.05.

Few days after isolation, the cells from umbilical vein exhibited morphological features of endothelial cells, including whirlpools or concentric circles of cells clones. By immunofluorescence, these cells showed positive expression of factor VIII (Fig. 2a). We then investigated how endothelial produced chemokines, like CCL22, CCL17.

Primary HUVECs were stimulated by normal or psoriatic sera, the latter of which had higher mRNA levels of both CCL17 and CCL22 than the former group (Fig. 2b). Furthermore, the TNF- α treatment exhibited enhancement effect on mRNA expression of CCL17, CCL22 in HUVECs (Fig. 2c), which was similar to psoriasis sera. Our data showed that serum from psoriasis patients or TNF-α promoted the protein expression of CCL17 and CCL22 in HUVECs while serum from normal in culture medium silenced the expression of these chemokines (Fig. 2d, Supplementary Fig. S2). Together, these results implied that TNF-α derived from the serum of psoriasis patients has the potential to induce chemokines from endothelial cells.

### TNF-α promoted expression of chemokines in HUVECs via activating ERK and JNK pathways

**Figure 3:**
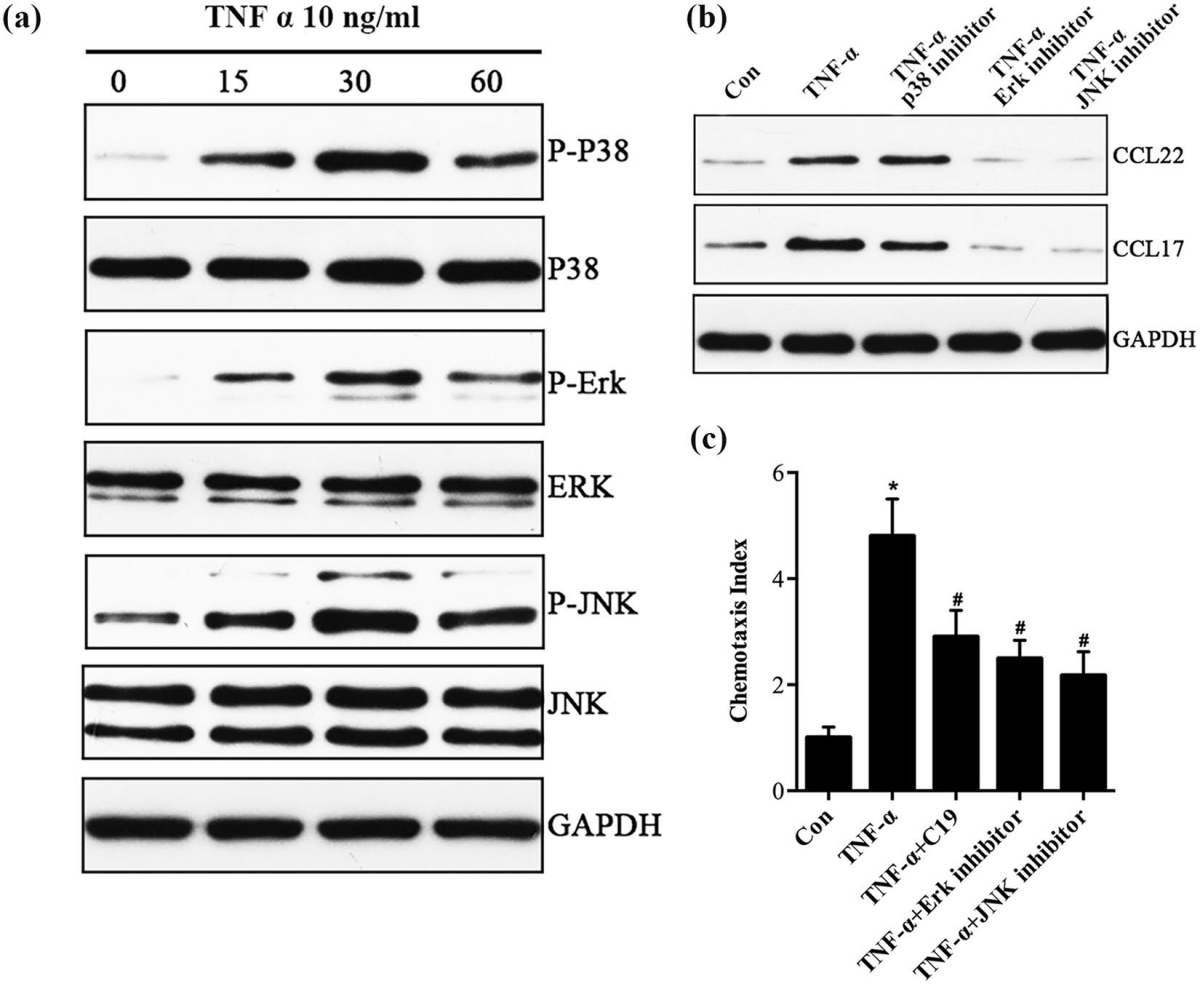
Effects of TNF-α on activation of p38-MAPK, ERK1/2 and JNK in HUVECs. (**a**) HUVECs were treated with TNF-α for 15, 30, and 60 mins, and the phosphorylation of p38-MAPK (p38^Thr180/Tyr182^), ERK1/2 (ERK1/2^Thr202/Tyr204^), and JNK (JNK^Thr183/Tyr185^) were detected by western blot. (**b**) HUVECs pre-treated with signal pathway inhibitors for 2 h, and incubated with TNF-α for 24 h. The expression of chemokines was detected by western blot. GAPDH was used as a loading control. (**c**) The transwell assay showed that C19 could inhibit chemotaxis of T cells. *p < 0.05 vs. control group; ^#^p < 0.05 vs. TNF-α group.

Although TNF-α may mediate chemokines production, the precise mechanism underlying this effect is still unknown. It is reported that the MAPK signaling pathways may be involved in TNF-α-mediated chemokines production^19,20^. To determine the potential involvement of protein kinase pathways in TNF-α-mediated chemokines production in HUVECs, we evaluated phospho-JNK, phospho-p38 MAPK and phospho-ERK1/2 expressions in HUVECs by western blot. Prominent increases in all these three MAPK levels were observed in HUVECs after stimulation with 10 ng/ml TNF-α at 15 mins to 30 mins (Fig. 3a, Supplementary Fig. S3). To elucidate the roles of these three MAPK signaling pathways in chemokines production from endothelia cells, ERK1/2 inhibitor (PD98059), JNK inhibitor (SP600125) and p38-MAPK inhibitor (SB2219) were used to treat HUVECs in the presence of TNF-α. PD98059 and SP600125 significantly attenuated TNF-α-mediated up-regulation of chemokines in HUVECs. In contrast, SB2219 had little influence on the expression of chemokines induced by TNF-α (Fig. 3b, Supplementary Fig. S3). These experiments revealed that the ERK1/2 and JNK MAPK signaling pathways might be the predominant signaling pathways in TNF-α-mediated vascular chemokines production.

### C19 inhibited chemotaxis of T cells

It was previously demonstrated that Jurkat cells, an immortalized line of human T lymphocytes, constitutively expressed CCR4 at the cell surface^21,22^. To determine the effect of C19 on the CCL17, CCL22 and CKLF1/CCR4 axis, we investigated the role of C19 in the chemotaxis of Jurkat cells following exposure to chemokines of TNF-α-mediated production. As shown in Fig. 3c, after stimulation with TNF-α, a large number of Jurkat cells migrated into lower well, and pretreatment of Jurkat cells with C19 attenuated the chemostaxis of Jurkat cells into lower well. In addition, HUVECs pretreated with PD98059 and SP600125 also attenuated the chemotaxis of Jurkat cells migrating into lower well. These results indicated that C19 could suppress chemotaxis of T cells to HUVECs, and ERK1/2, JNK MAPK signaling pathways might be involved in TNF-α-induced T cell chemotaxis to HUVECs.

### Antagonistic effect of CKLF1-C19 on proliferation of HUVEC through ERK1/2 and p38-MAPK signaling pathways induced by CKLF1

**Figure 4:**
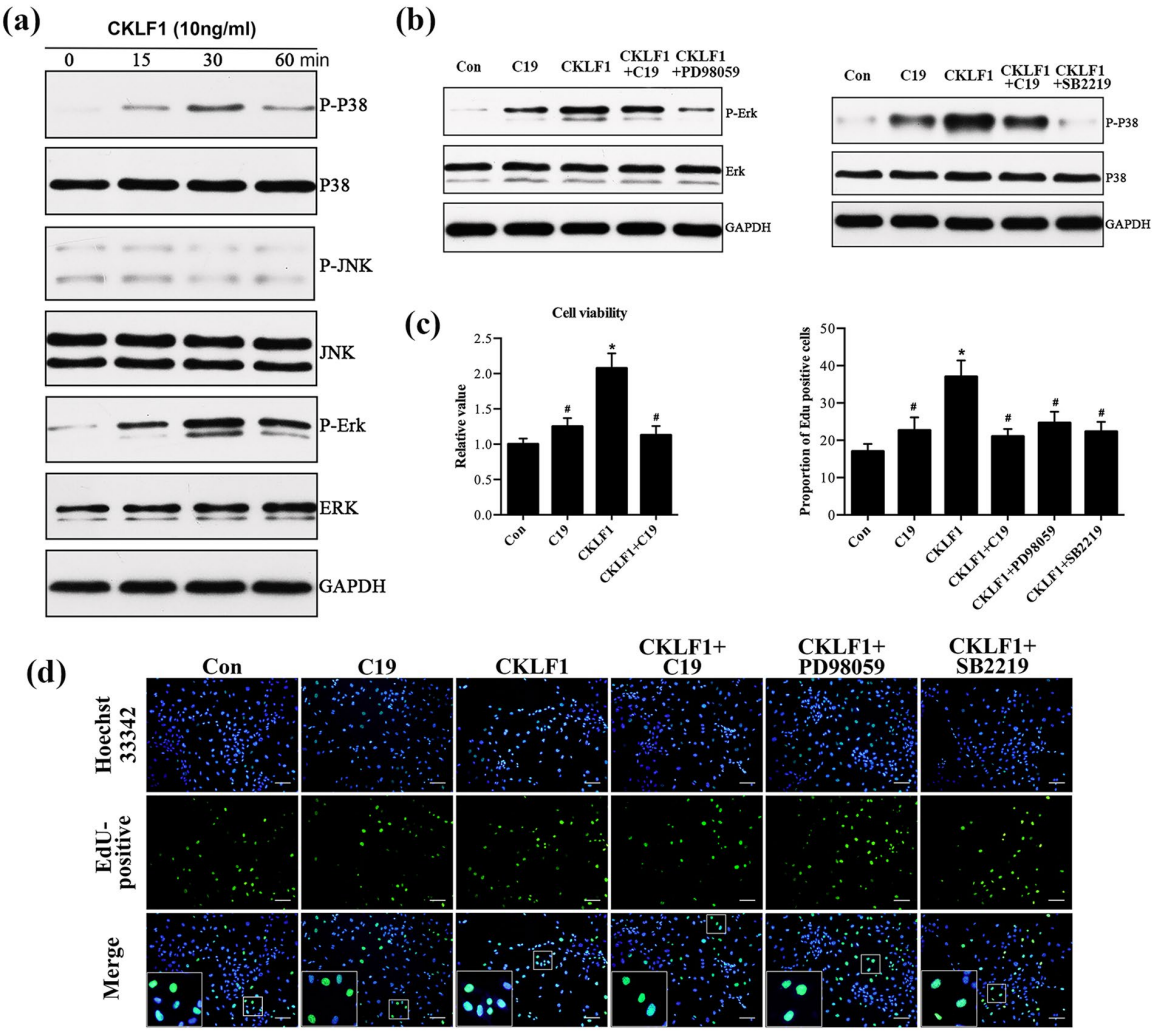
CKLF1-C19 attenuated the proliferation of HUVEC and MAPK pathway activity induced by CKLF1. (**a**) HUVECs were treated with CKLF1 for 15, 30, and 60 mins, and the phosphorylation of p38-MAPK, ERK1/2, and JNK were detected by western blot. (**b**) HUVECs pre-treated with signal pathway inhibitors for 2 h or C19 for 1 h, and incubated with CKLF1 for 30 mins. The activity of signaling pathway was detected by western blot. GAPDH was used as a loading control. (**c**) The CCK-8 assay showed that pretreatment with of C19 could reduce CKLF1-mediated HUVECs proliferation, which was consistent with the results of EDU (**d**). Scale bar = 100 μm. *p < 0.05 vs. control group; ^#^p < 0.05 vs. CKLF1 group.

The precise involvement mechanisms effects of CKLF1 in psoriasis have not been established. According to Kong *et al*.^16^, the MAPK signaling pathway might be involved in CKLF1-mediated inflammation of focal cerebral ischemia. To explore the potential involvement of protein kinase pathways in CKLF1-mediated proliferation, we detected phospho-JNK, phospho-p38 MAPK and phospho-ERK1/2 in these cells using western blot. A prominent increase of phospho-ERK1/2 and p38-MAPK level was observed in HUVECs after stimulation with CKLF1 (10 ng/ml) (Fig. 4a, Supplementary Fig. S4). Additionally, the decreased expression of phospho-ERK1/2 and phospho-p38 were detected when cells were exposed to CKLF1-C19 (100 ng/ml) for an hour prior to CKLF1. Moreover, SB 2219 and PD98059 could weaken the expression of phospho-p38 and phospho-ERK1/2 MAPK (Fig. 4b, Supplementary Fig. S4). These experiments revealed that the p38 and ERK1/2 MAPK might be important signaling pathways in C19-mediated antagonism effect on endothelial cells hyperplasia induced by CKLF1.

To further confirm whether the inhibition of the signaling was by C19 and whether C19 would shorten cellular life span, the effects of C19 on the growth kinetics of HUVECs in the present of CKLF1 were assessed using cell counting kit-8 (CCK-8). As shown in Fig. 4c, C19 could reduced the growth ability of HUVECs induced by CKLF1. Furthermore, the EdU proliferation assay (Fig. 4d) demonstrated similar results: less EdU-positive cells were in the preprocessed C19 group than in the CKLF1 group.

To elucidate the role of the ERK1/2 and p38-MAPK, PD98059 and SB 2219 were used to treat HUVECs in the presence of CKLF1. PD 98059 and SB 2219 significantly attenuated CKLF1-mediated upregulation of proliferation (Fig. 4d). These results indicated that ERK1/2 and p38 signaling pathways might play key roles in CKLF1-C19-mediated inhibition of endothelial cell proliferation, which was induced by CKLF1.

### CKLF1-C19 considerably ameliorated imiquimod-induced psoriasiform inflammation (IPI)

**Figure 5:**
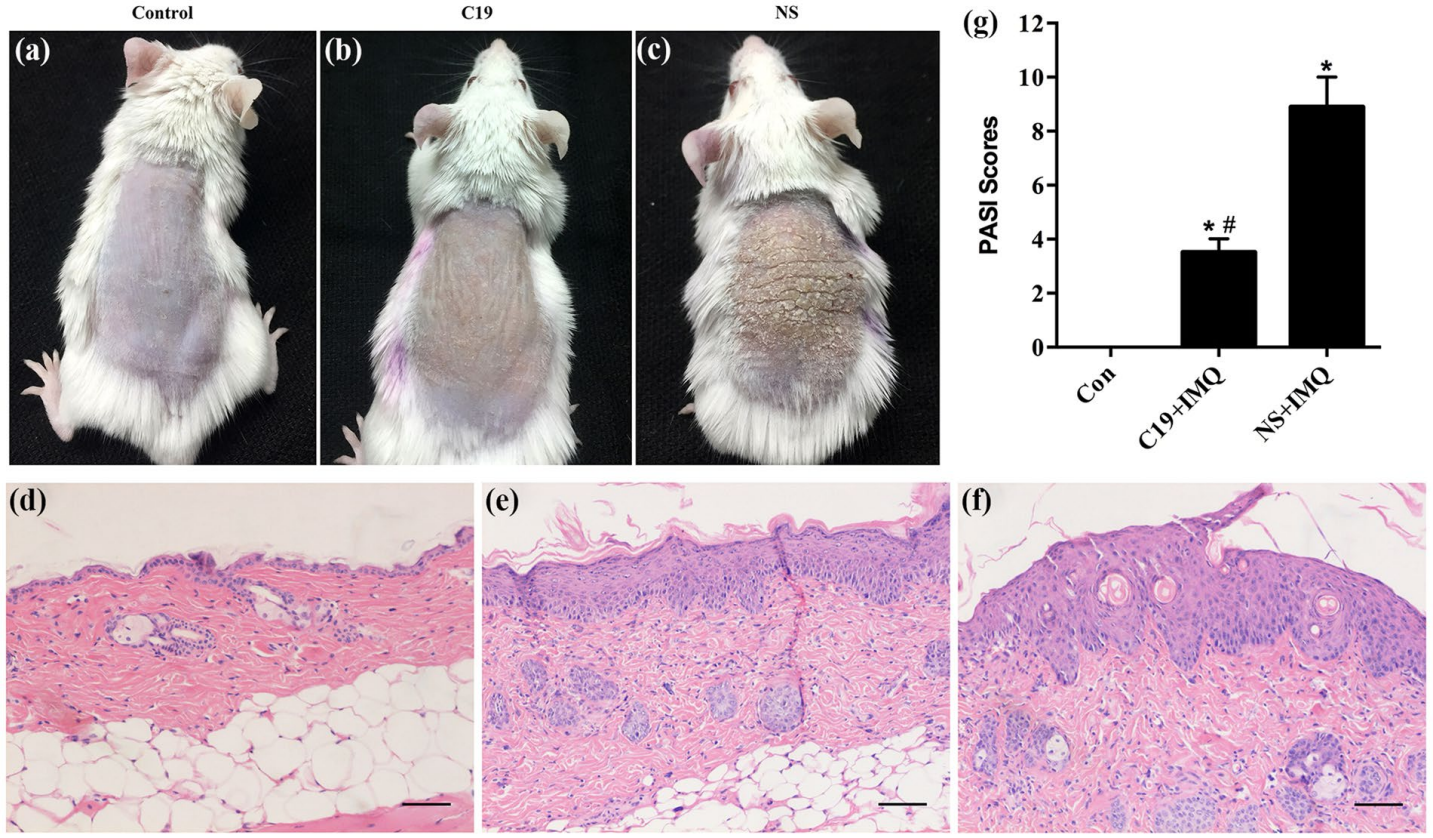
CKLF1-C19 significantly attenuated IPI. (**a–c**) Respective clinical presentations of control group mice, IPI of mice from CKLF1-C19 group and NS group. (**d–f**) Respective histopathological changes of lesions from control group mice, C19 group mice and NS group mice (H&E, original magnification 200x ). (**g**) Obvious hyperkeratosis, acanthosis and inflammatory infiltrates were observed in IPI of NS group mice. The histogram indicated the average adjusted PASI score of C19 group mice was significantly lower than that of NS group mice (p < 0.01). Scale bar = 50 μm. *p < 0.05 vs. control group; #p < 0.05 vs. NS + IMQ group.

BALB/c mice injected intradermal with normal saline (NS) or C19 had imiquimod (IMQ) cream or simple cream base of IMQ (vehicle) topically applied to the shaved back for 6 consecutive days. The control group mice had no observable changes in the local skin (Fig. 5a). The results indicated that the vehicle caused no irritant dermatitis, the mice in the NS group had erythema, scales and incrassation of the back skin from day 3 onward, which became most obvious on day 6 (Fig. 5c). However, the mice in the C19 group showed erythema, scales and incrassation from day 4 onward, which was less severe in contrast to that of NS group mice at the end of experiment (Fig. 5b). In the lesional skin of the NS group mice, histopathological changes mainly included hyperkeratosis, acanthosis and inflammatory infiltrates in the dermis resembling human psoriasis (Fig. 5f). C19 ameliorated these changes obviously but did not clear them completely (Fig. 5e). Since psoriasiform inflammation was induced by topical application of IMQ cream, the Psoriasis Area and Severity Index (PASI) of this model was adjusted according to the extent of erythema, scales and thickness from grade 1–5 in correspondence to 0–4 scores^23^. The average adjusted PASI in the C19 group mice was much lower than that in NS group mice (Fig. 5g). Also, clinical presentations and histopathological changes of C19 group mice were still obvious compared to those of control group.

### C19 reduced infiltration of inflammatory cells and dermal endothelial cells

**Figure 6:**
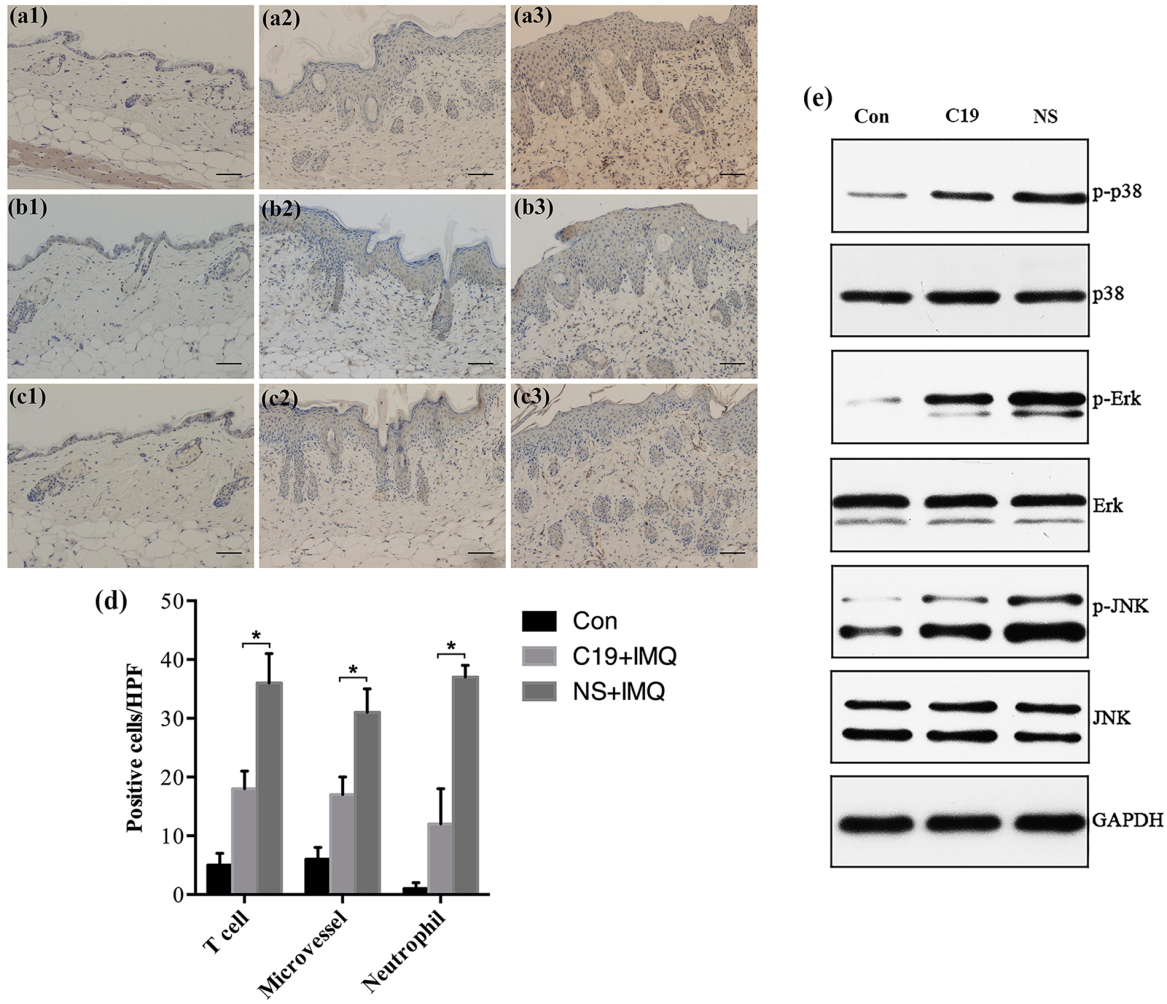
Infiltration of inflammatory cells and dermal microvessels were reduced along with amelioration of IPI by CKLF1-C19 via MAPK. Sections from back lesional skin were analyzed by immunohistochemistry using the markers CD3 (T cell), MPO (neutrophil) and CD31 (endothelial cell). (**a1-c1**) Respective stainings of T cells, endothelial cell and neutrophils of lesions from control group; (**a2-c2**) respective stainings of C19 group mice; (**a3-c3**) respective stainings of normal saline (NS) group. Histogram (**d**) indicated the numbers of positive cells per HPF of C19 group mice was significantly less than that of NS group mice. (**e**) The effect of CKLF1-C19 on MAPK signal pathways in IPI whole skins. Number of mice was five in each group. Scale bar = 50 μm. *p < 0.05.

Both T cells and neutrophils play important roles in the pathogenesis of psoriasis. The erythema of psoriasis is due to a great number of dilated dermal blood vessels. Thus, we used immunohistochemistry to analyze the effects of C19 on the infiltration of T cells, and neutrophils and proliferation of endothelial cell in lesional skin. Along with the amelioration of IPI in C19 group mice, the dermal infiltrations of T cells, neutrophils and dilatation of microvascular were reduced significantly compared with those of NS group mice (Fig. 6a–c).

### C19 inhibited the activation of MAPK signal pathways

Mitogen-activated protein kinase (MAPK) signal transduction pathways including p38, ERK and JNK are the most important signaling molecules thought to mediate the inflammatory response^24–26^. Six days after IMQ applied, the phosphorylation level of p38, ERK and JNK was increased significantly in the NS group (Fig. 6e, Supplementary Fig. S5). Treatment with C19 at dose of 10 μg could significantly decrease the phosphorylation level of p38, ERK and JNK (p < 0.01).

## Discussion

Our study demonstrated that pretreatment with C19 could decrease the infiltration of inflammatory cells and proliferation of microvascular cells *in vivo* and *in vitro*, indicating the protective role of C19 in psoriasis.

A preponderance of evidence from clinical and experimental studies suggested that chemokines were implicated in a wide range of disease with prominent inflammatory components, such as rheumatoid arthritis, asthma, multiple sclerosis and arteriosclerosis^27,28^. Cytokines and chemokines play crucial roles in the transendothelial migration of inflammatory cells such as neutrophils, monocytes/macrophages, and lymphocytes. In the present study, administration of CKLF1-C19 decreased the number of MPO-positive neutrophils, which was a marker enzyme for measuring neutrophils accumulation in psoriatic lesions. In addition to neutrophils, other leukocyte subsets were also involved in psoriasis-related immune-inflammation. More specific, in the case of psoriasis, where T-cell-mediated autoimmune skin disease has been identified^29^, dermal endothelium are key participants in innate immunity recruiting T cells, such as Th1, Th17 and Th22 to the skin, and T cells are important in sustaining disease activity. Some proinflammatory mediators, such as cytokines and chemokines, are up-regulated in psoriatic lesions, animal models of psoriasis and their inhibition or deficiency has been associated with reduced clinical appearance^30–32^. CCR4 plays a role in the recruitment of the memory T lymphocyte to the skin together with CCR10, which express on memory T cells. CCR4 is accompanied by CCL17, a chemokine secreted by epidermal Langerhans cells, fibroblasts, and blood endothelial cells^11,33^. CCR4 and CXCR3 receptors are highly expressed on leukocytes which infiltrate the psoriatic lesions, while in the dermis a high expression of their ligands is found: CCL17 and CCL22 together with CXCL9 and CXCL10. CCR4, as a functional receptor for CKLF1, prevalently expressed on T cells, and its ligand CCL17 and CCL22 are involved in lymphocyte-endothelial interactions during lymphocyte recruitment to normal and inflamed skin^11^. Therefore, the chemokines, modulating immune process in psoriasis, are potential targets in future psoriasis treatment^9^.

As a novel member of the CC chemokine family, CKLF1 has multiple activities, including chemotactic activities in a wide spectrum of leukocytes, an ability to stimulate the migration of vascular smooth muscle cells^34^, and the higher expression of CKLF1 during T lymphocyte activation^12^. Over-expression of CKLF1 in mice caused dramatic pathological changes in lungs that are similar to those observed in human asthma. These findings implied that CKLF1 might play important roles in the inflammatory response. As a secreted form of CKLF1 located in C-terminal part, C19 can weakly activate CCR4 signal^14,15^. It had been proven in our previous study that C19 could promote cell proliferation via CCR4^18^. Additionally, the CKLF1 peptides function through the identical receptor of CCR4. Meanwhile, reported studies also showed that CKLF1 was associated with neuronal apoptosis but not proliferation^35^. However, the C19 was reported to protect against focal cerebral ischemia and allergic lung inflammation and inhibit leukocyte chemotaxis which were the results of CKLF1 activation^16,17,21,36,37^, a potential candidate of antagonist peptide of CCR4. The difference between the cells induced by CKLF1 and C19 might be related to the differential origins of cells. Actually, the same chemokines might induce diversities of biological effects, which could also be the results of differences in local inflammatory environment^38,39^. However, the protective role of C19 in psoriasis remained unknown. Therefore, the defensive effect of C19 in psoriasis was investigated. The results of the present study showed that pretreatment with C19 inhibited the infiltration of inflammatory cells and proliferation of microvascular cells. These results, together with the previous findings that the expression of CKLF1 was induced in the psoriasis lesions, suggesting that the CKLF1 produced in the skin played a crucial role in this immune-related disease. In addition, because C19 is a peptide derived from human CKLF1, there is a decreased chance of immunoresponse in human body. Chemical synthesis of C19 peptides satisfies the demand for its large-scale production. Therefore, C19 is an ideal candidate for application as a peptide drug for the treatment of psoriasis.

TNF-α and psoriatic patients’ serum led to the production of chemokines, CCL22, CCL17 and CKLF1 in endotheliocyte, in both mRNA level and protein level. CCR4 on T cells and the ligands CCL17, CCL22 and CKLF1 might also play an important role in lymphocyte trafficking to psoriatic skins. In this study, we used immunohistochemistry to localize CCL17 expression to the dermal endothelium in inflamed skins. Thus, these chemokines might be essential to activate CCR4+ lymphocytes tethered to the dermal blood vessels and mediate trans-endothelial migration. Collectively, these data suggested that CCR4 and ligands may be involved in T cell recruitment to the inflamed dermis. According to the results of transwell and immunohistochemistry of CD3+ T cells in mice, we propose that C19 peptide protects against psoriatic inflammation by decreasing chemotaxis of T cells induced by CKLF1, CCL17 and CCL22.

Vascular endothelial cells are also closely linked to psoriatic disease because the inflammatory milieu leads to induction and activation of a range of pro-angiogenic factors^40^. HUVECs are widely used as a source of endothelial cells and are extrapolated to dermal microvascular endothelial cells in psoriatic dermis^41,42^. Moreover, the TNF-α-governed pro-inflammatory environment in psoriatic skins induces endothelial chemotactic molecules, which facilitates the recruitment of circulating leucocytes^43^. In this study, the endothelial cells isolated from freshly umbilical cords displayed the features of HUVECs, including typical morphology and positive expression of VIII factor. Therefore, the changes of these cells reflected the status of dermal microvascular endothelial cells in psoriasis. To identify the antagonistic effect of C19 on angiogenesis formation, C19 was used to pretreat endothelial cells in both animal model and cellular model. The present study demonstrated that the ratio of proliferation of endothelial cell was significantly decreased compared to CKLF1 group. In addition, it was observed that C19 rivalry microvascular changes *in vivo*, including dilation and endothelial cell proliferation, further confirming the role of C19 in protection against the development of psoriasis.

The MAPK represents important regulatory signaling molecules that serve as integration points connecting extracellular signals to the transcriptional programs of the cell. Studies on the relationship between the MAPK pathways activation and psoriasis demonstrated that MAPK, including p38 (p38 MAPKs), ERK1/2, and JNK expression, were greatly increased in lesional psoriatic skin^44^. The MAPK controlled several important functions within the cell, such as cell proliferation, differentiation, gene expression, and apoptosis^45^. Some studies indicated that inhibition of the MAPK pathways reduced TNF-α, IL-8 expression and protected against psoriasis^26,46^. Our *in-vitro* experiment demonstrated that TNF-α promoted the expression of chemokines as CCL17 and CCL22 in HUVECs via activating ERK, JNK pathways and the antagonistic effect of CKLF1-C19 on CKLF1 induced proliferation of HUVECs was through ERK1/2 and p38-MAPK signaling pathways. In this animal model, treatment with C19 inhibited the phosphorylation level of p38, ERK and JNK in the psoriatic lesion. Therefore, C19 could affect the MAPK signal pathways to protect against psoriatic lesion.

In conclusion, this study shows that pretreatment with C19 can significantly protect against psoriasis by decreasing chemotaxis of T cells and neutrophils, reducing the proliferation of endothelial cells and attenuate IPI of BALB/c mice, improving clinical presentations broadly, possibly via inhibiting MAPK signal pathways. Therefore, CKLF-C19 may be a novel direction for the treatment of psoriasis.

## Methods

### Ethical statement

The study was approved by the Ethics Committee of Beijing Chaoyang Hospital and conducted according to the Declaration of Helsinki. Written informed consent was obtained from all patients before study. All animal experiments were performed according to the Guide for the Care and Use of Laboratory Animals and the experimental protocols were approved by the Laboratory Animal Center, Beijing Chaoyang Hospital.

### Tissue samples

We obtained skin biopsies of psoriatic lesions from 15 outpatients (9 males and 6 females, mean age 41.3 years; skin involvement 16–40%) at Beijing Chaoyang Hospital, who had been diagnosed both clinically and pathologically. Biopsy tissues were taken from center of a plaque in patients with untreated moderate-to-severe psoriasis vulgaris. The site-, gender-, and age-matched normal skins were collected from the patients undergoing routine surgery but without cutaneous or inflammatory-mediated diseases. PBMCs samples were also harvested from these patients accordingly.

### Mouse model of IPI

Eight-week-old BABL/c mice were purchased from the Animal Department of Capital Medical University and were housed in a specific pathogen free facility. The housing conditions were controlled, with a temperature of 20–25 °C and a 12:12 hours light: dark cycle. Mice were numbered and randomly assigned to three groups, then, an area of 5 cm × 3 cm was shaved from the backs of all mice. Mice in the control group were given IMQ base cream topically and then intradermally injected with 100 μl normal saline, while mice in the normal saline (NS) group and C19 group received a daily topical application of 62.5 mg IMQ cream (5%, Aldara, 3 M Pharmaceuticals, Shanghai, China), followed by intradermal injection of 100 μl normal saline without or with 10 μl C19 peptide (Hybio Engineering, Shenzhen, China) for 6 consecutive days. At the end point of the experiment, all mice were killed by breaking the neck. Samples of skin from back lesions were collected for latter experiments.

### Immunohistochemistry

Tissues were processed and embedded in paraffin using routine methods. Tissue blocks were serially sectioned to obtain consecutive levels. Sections were stained with H&E, and immunohistochemistry was performed as previously described^18^ by using antibodies against CCL17, CD3, CD31 and MPO (all from Abcam, Cambridge, MA, USA).

### Immunofluorescence

Fluorescent detection was performed on frozen sections. After heat-mediated antigen retrieval as above, slides were incubated in blocking buffer (10% goat serum in PBS) for 1 hour. After washed with PBS, slides were hybridized at 4 °C overnight with rabbit anti-human CCL17 and mouse anti-human CD31 antibodies. The detection antibodies were fluorescein isothiocyanate-labeled goat anti-rabbit and tetraethyl rhodamine isothiocyanate-labeled rabbit anti-mouse antibodies (Southern Biotech, Birmingham, AL, USA). Nuclei were counter stained with Hoechst 33342. Sections were then imaged using the type BM-LB2 fluorescence microscope (Leica, Bensheim, Germany).

### Cell culture and treatment

HUVECs were prepared using a previously described method^47^. Briefly, umbilical cord veins were cannulated and flushed with PBS to remove blood and then filled with 0.25% trypsin for 15 mins at 37 °C. Isolated endothelial cells were maintained in Medium 200 containing 10% fetal bovine serum (Cascade Biologics, Mansfield, UK), 2% low serum growth supplement. Cultured cells were identified as endothelial cells based on their morphology and the expression of VIII factor by immunofluorescence^48^. Jurkat cells (kindly provided by Dr. Xinghua Xu from the Chinese PLA General Hospital, Beijing, China) was cultured in RPMI-1640 medium supplemented with 10% FBS. All cells were cultured at 37 °C with 5% CO_2_.

Endothelial cells were seeded in plates to 90–100% confluence and then exposed to recombinant human TNF-α (Abcam) or CKLF1 (obtained from Peking University Center for Human Disease Genomics, Beijing, China) for 0 min, 15 mins, 30 mins, and 60 mins. Celluar protein was harvested, phosphorylation status of the three major protein kinases (JNK, ERK, p38) was surveyed by western blot.

To explore the level of expression of CCL17 and CCL22, and possible related signaling pathways, the endothelial cells were seeded in 6-well plates at a density of 2 × 10^6^ cell/well and cultured overnight. Then, TNF-α was added into the meduim at a final concentrations of 10ng/ml or additionally supplemented with 10% psoriasis sera. The cells were incubated with TNF-α or psoriasis sera for 24 h before undergoing qRT-PCR and western blot to analyze the expression of CCL17 and CCL22.

PD98059, SP600125, and SB2219 (all from Target Molecule, Boston, USA) were used to identify the downstream signaling mediators of TNF-α and CKLF1. All these inhibitors were dissolved in dimethylsulfoxide (Invitrogen, Grand Island, NY, USA) at a concentration of 50 mmol/L and added to fresh culture medium to achieve the desired final concentration of 20 μmol/L. Cells were pretreated with these three inhibitors for 2 h, and then incubated with TNF-α or CKLF1.

To determine whether C19 could decrease the endothelial cells proliferation and viability, we pretreated the cells with 100 ng/ml C19 for an hour and then incubated with 10 ng/ml CKLF1.

### CCK-8

Following the manufacturer’s instructions, CCK-8 (Dojindo, Kumanoto, Japan) was used to measure the viability of HUVECs. First, 5 × 10^3^ cells were plated in a volume of 100 μl into each well of 96-well plates. They were given various stimulations for 24 h. When all wells were treated as indicated, 10 μl CCK-8 was added to each cell culture insert and incubated for 3 h at 37 °C, 5% CO_2_. After incubation, an orange soluble formazan product was formed. The formazan product was spectrometrically quantified using an Elisa reader (Tecan, Maennedorf, Switzerland). All assays were performed in triplicate.

### EdU assay

EdU assay (RIBOBio Co, Guangzhou, China) was used to measure cells’ abilities to proliferate after above treatment for 24 h. After incubation with EdU for 4 h, the cells were fixed with 4% paraformaldehyde and permeabilized with 0.5% Triton X-100. Then, the Apollo reaction cocktail (reaction buffer and Apollo® 643 fluorescence) was added to medium for another 30 mins in the dark. After washed with PBS for three times, and the nuclei were stained with Hoechst 33342 and immediately viewed under fluorescence microscopy. Cell proliferation ratios were calculated using the formulation of (Edu-positive cells/Hoechst-stained cells) × 100%. The number of EdU-positive cells were calculated by counting at least three random separate fields.

### qRT-PCR

Total RNA of fresh tissues or cell cultures was purified with Trizol reagent (Invitrogen, Grand Island, NY, USA). cDNAs were synthesized by using cDNA kit (Applied Biosystems, Carlsbad, CA, USA), and mRNA expression was determined by using a SYBR Green Master Mixes (Thermo Fisher Scientific, Waltham, MA, USA). The sequences of each primer pair and the product size are presented in Table 1. The 2^−ΔΔC^_T_ method was used to normalize transcription to GAPDH and to calculate the fold induction relative to controls. qRT-PCR was performed in triplicate, including non-template controls.

**Table 1:** Primer pairs used for qRT-PCR analysis.

Gene	Forward (5′-3′)	Reverse (5′-3′)
GAPDH	AAGGTGAAGGTCGGAGTCAAC	GGGGTCATTGATGGCAACAATA
CCR4	CCATCTCGGATCTGCTCTTC	AGCCCACCAAGTACATCCAG
CCL17	AGGGAGCCATTACCCTTAGA	CTCTTGTTGTTGGGGTCCGA
CCL22	ACTGCACTCCTGGTTGTCCT	GCAGACGGTAACGGACGTA

### Western blotting

Proteins of HUVECs or fresh tissues were prepared for western blot analysis. Protein concentration of each sample was quantified with the BCA protein assay (Beyotime Institute of Biotechnology, China). Then, equal amounts of proteins were separated by electrophoresis on 10% polyacrylamide gels and transferred to polyvinylidene difluoride membranes. The membranes were blocked with 5% milk in Tris-buffered saline and 0.2% Tween at room temperature for 1 h and then incubated overnight at 4 °C with the following specific primary antibodies: GAPDH (1:1000, Cell Signaling Technology), ERK (1:1000, Cell Signaling Technology), p-ERK (1:1000, Cell Signaling Technology), p38 (1:500, Abcam), p-p38 (1:1000, Cell Signaling Technology), JNK (1:500, Santa Cruz Biotechnology), p-JNK (1:500, Santa Cruz Biotechnology), CCL17 (1:1000, Abcam), and CCL22 (1:1000, Abcam). The blots were washed three times with Tris-buffered saline and 0.2% Tween and incubated with a horseradish peroxidase-conjugated secondary antibody (Santa Cruz Biotechnology) for 45–60 min at room temperature. The expression signals were detected with an enhanced chemiluminescence reagent (Beyotime Institute of Biotechnology, China) after the membranes were washed with TBST (10 min × 3).

### Chemotaxis assay

The chemotaxis assay was performed using a 24-well transwell chamber with a pore size of 5 μm (Corning, USA). First, 10^5^ HUVECs were seeded in the lower wells (600 μl/well), pretreated with or without signaling pathway inhibitor, and then incubated with TNF-α (10 ng/ml) for 24 h. Then cells were resuspended in the same medium at 10^6^ cells/ml and added to the upper wells (100 μl/well), which were seperated from the lower wells by polyvinylpyrrolidone-free polycarbonate filter with 5 μm pores for Jurkat cells. The chamber was incubated for 2 h for Jurkat cells at 37 °C in 5% CO_2_. Jurkat cells that had migrated into the lower well were counted by cell counting plate. The chemotaxis index was calculated as the number of cells that migrated to the sample medium divided by the number of cells that migrated to the control medium. Cells were pretreated with indicated factors for 1 h at 37 °C before they were loaded into the upper chamber.

### Statistical analyses

The data were presented as mean ± standard deviation (SD). The two-tailed t-test was used to compare continuous data between two groups. For comparisons of continuous data among multiple groups, one-way analysis of variance (ANOVA) was performed. Statistical analysis was assessed using SPSS 22.0 software (SPSS Inc., Chicago, USA). All statistical tests were two-sided. Values of p < 0.05 were considered statistically significant.

## Electronic supplementary material

Supplementary Figures
