# Supplementary material for: C19, a C-terminal peptide of CKLF1, decreases inflammation and proliferation of dermal capillaries in psoriasis
Source: Sci Rep. 2017 Oct 24;7:13890. doi: 10.1038/s41598-017-13799-x (PMC5655640; doi:10.1038/s41598-017-13799-x)
Supplement: Supplementary file 1 — Supplementary Figures [file 41598_2017_13799_MOESM1_ESM.pdf]

C19, a C-terminal peptide of CKLF1, decreases inflammation and proliferation of dermal capillaries in psoriasis

Yi Zheng, Yixuan Wang, Xuan Zhang, Yaqi Tan, Shiguang Peng, Le Chen and Yanling He

Department of Dermatology, Beijing Chaoyang Hospital Affiliated to Capital Medical University, Beijing, China

Correspondence to: Yanling He, email: dermhe@163.com

Supplementary Figure S1. CCL17 protein expression in normal and psoriatic lesions.

Supplementary Figure S2. Uncropped gel image for Figure 2.

Supplementary Figure S3. Uncropped gel image for Figure 3.

Supplementary Figure S4. Uncropped gel image for Figure 4.

Supplementary Figure S5. Uncropped gel image for Figure 6.

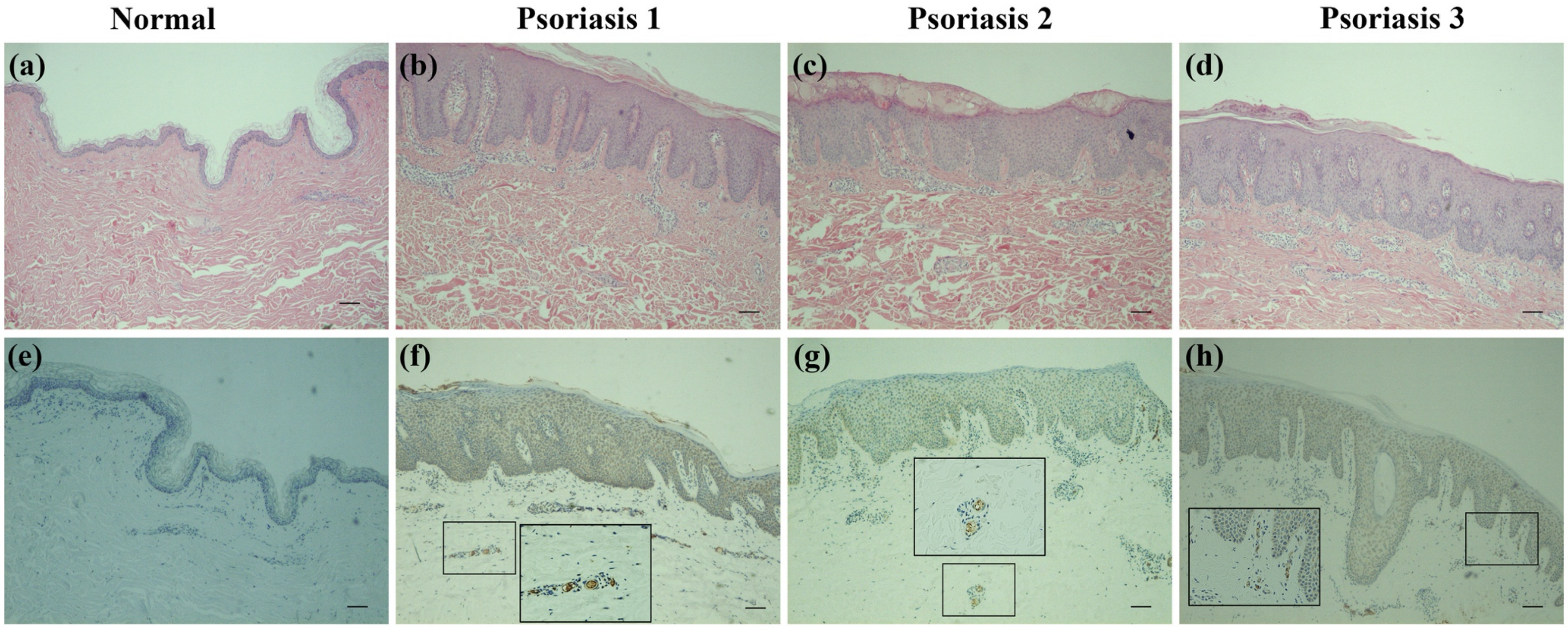

Supplementary Figure S1. CCL17 protein expression in normal and psoriatic lesions.

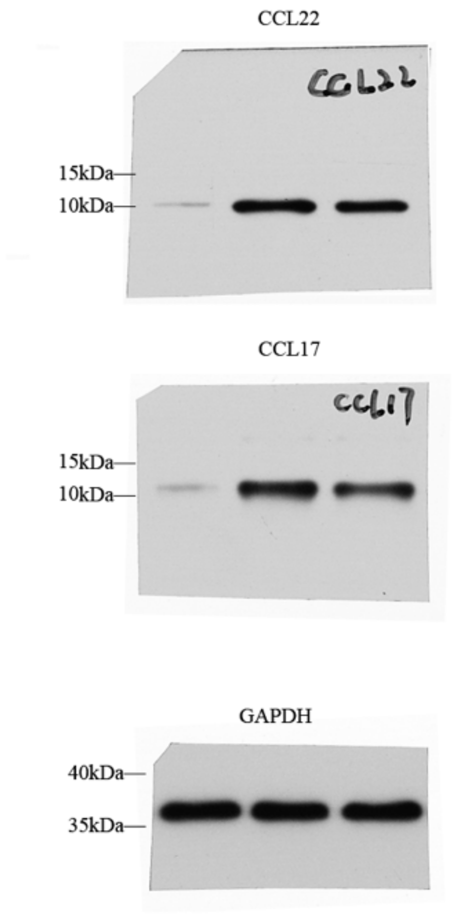

Supplementary Figure S2. Uncropped gel image for Figure 2.

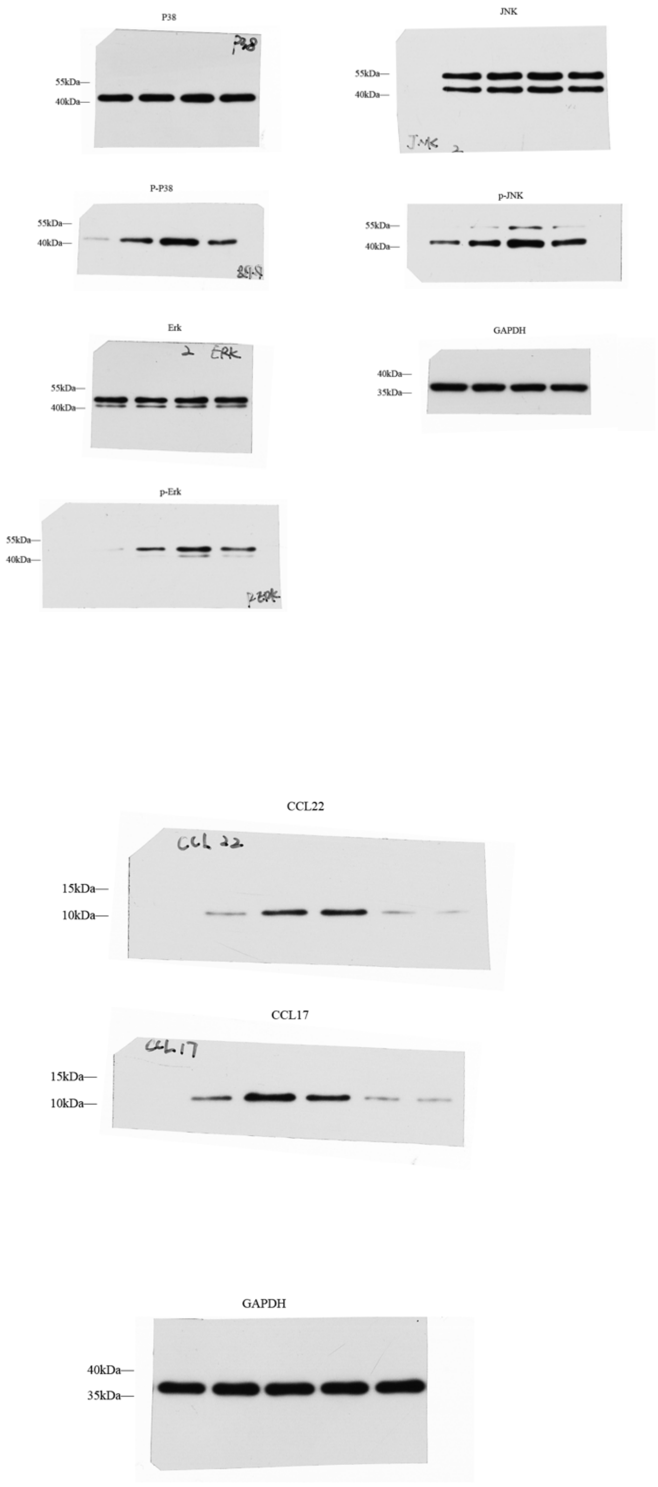

Supplementary Figure S3. Uncropped gel image for Figure 3.

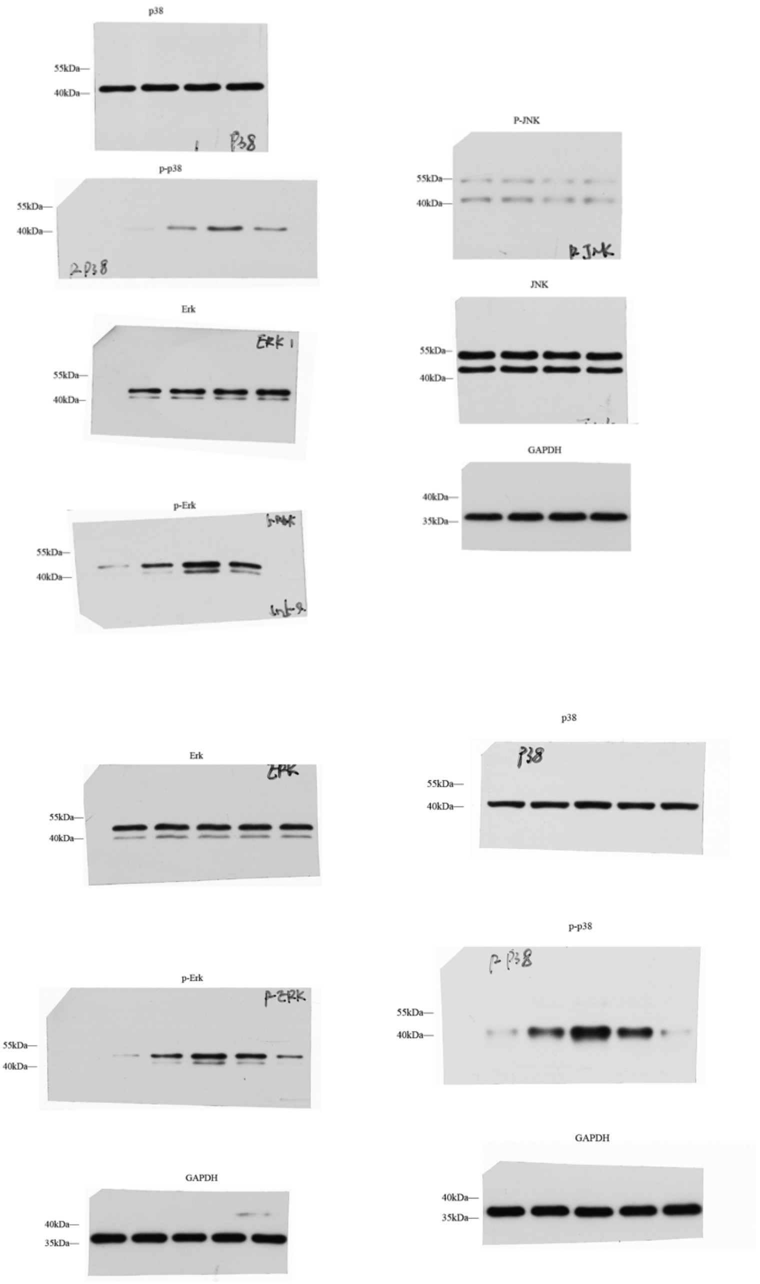

Supplementary Figure S4. Uncropped gel image for Figure 4.

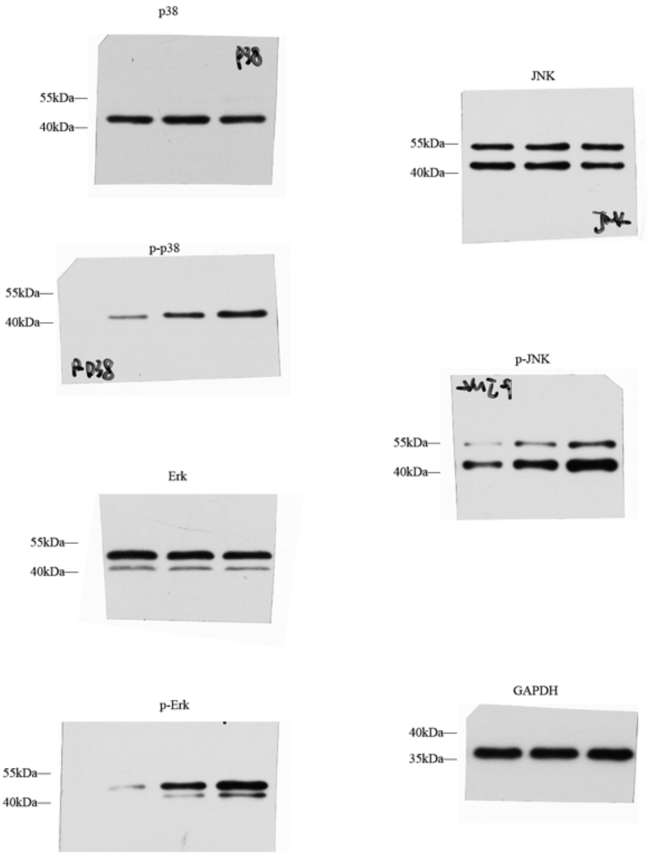

Supplementary Figure S5. Uncropped gel image for Figure 6.
